# “Everywhere but not specifically somewhere”: a qualitative study on why the right to health is not explicit in the post-2015 negotiations

**DOI:** 10.1186/s12914-015-0061-z

**Published:** 2015-08-21

**Authors:** Claire E. Brolan, Peter S. Hill, Gorik Ooms

**Affiliations:** The School of Public Health, Faculty of Medicine and Biomedical Science, The University of Queensland, Herston Road, Herston, Brisbane, Queensland 4006 Australia; Department of Public Health, Institute of Tropical Medicine, Nationalestraat 115, 2000 Antwerp, Belgium; Law and Development Research Group, Faculty of Law, University of Antwerp, Venusstraat 23, 2000 Antwerp, Belgium

**Keywords:** Right to health, Human rights, Global health, Qualitative research, Post-2015, Sustainable Development Goals

## Abstract

**Background:**

The Millennium Development Goals expire at the end of 2015 and global negotiations are underway to finalise the post-2015 Sustainable Development Goals. Much activism has occurred encouraging a post-2015 health and development goal embedded in the highest attainable standard of health (‘right to health’). Despite this, the right to health was absent in three key post-2015 intergovernmental Sustainable Development Goal proposals in 2014, one of which was reinforced by the United Nations General Assembly in September 2014 as the guiding document for ongoing interstate negotiations. This article examines why it appears the right to health, so far, is not gaining direct expression in post-2015 discussion.

**Methods:**

This qualitative research is part of a broader study using thematic and discourse analysis examining the high-level policy debate on health goals in the discourse of the formulation of the post-2015 Sustainable Development Goals. Key-informant interviews were conducted in two interview rounds in 2013 and 2014, with participants from multilateral and other organisations (government, academia, civil society and philanthropy) responsible for health in the post-2015 development agenda (or the post-2015 development agenda more broadly). This study synthesises data from both interview rounds on Health and Human Rights in post-2015 Sustainable Development Goal negotiations.

**Results:**

Six reasons why the right to health may not have gained effective traction in the unfolding post-2015 Member State negotiations were found. The first three reasons relate to broader issues surrounding human rights’ (including sexual and reproductive health and rights) positioning within international relations discourse, and the second three relate to the challenges of transforming the human right to health into a practically applied post-2015 health goal.

**Conclusions:**

This paper reports the views of participants, many of who sit at the interface of United Nations and Member State negotiations, on the right to health’s location (and projected trajectory) at two temporal junctions in evolving post-2015 negotiations. The interviews provide insight into high-level hesitancy that the right to health be expressly incorporated in the final post-2015 health and development goal, as well as documents participants’ doubt that rights language will explicitly frame the broader Sustainable Development Goals, their targets and indicators.

## Background

Global negotiations are in earnest to identify the new goals and targets for the Millennium Development Goals’ (MDGs’) successor, the post-2015 Sustainable Development Goals (SDGs). Despite activism encouraging a right to health goal [1–7], an explicit post-2015 health rights narrative is not gaining effective traction in intergovernmental post-2015 proposals. The purpose of this qualitative research, therefore, is to examine how or if the right to health is strategically intersecting in evolving post-2015 health and development dialogue. The right to health’s position in emerging post-2015 discussion is of import to the researchers who are part of the larger Goals and Governance for Health research team (or ‘Go4Health Project’). Go4Health is a consortium of academics and civil society members tasked with advising the European Commission on the international health-related goals to follow the MDGs, advocating “the right to health and its imperative of narrowing health inequities should be central to the post-2015 health agenda” [1]. This study thus draws on a discourse and thematic analysis of data obtained from two rounds of in-depth interviews, one immediately after the High-Level Panel of Eminent Persons on the Post-2015 Agenda’s (High-Level Panel) released its report in June 2013, and the second almost 12 months later in April-May 2014, with participants from key multilaterals and related agencies working on health in the post-2015 development agenda, many who sit at the interface of UN and Member State interaction. In finding the right to health is indeed at the periphery of post-2015 health and development dialogue, we provide six reasons participants gave for its marginalisation. We conclude by highlighting why the right to health’s lack of incorporation in the post-2015 metrics framework should raise concern for health rights advocates worldwide.

### Health’s Evolving Location in the Post-2015 SDG Agenda

In late May 2013 post-2015 SDG debate intensified when the High-Level Panel proffered 12 potential post-2015 goals in its report to the UN Secretary General [8]. One health goal – Goal 4 Ensure Healthy Lives – was tendered. Within the global health community, this submission cemented both anticipation and discourse around health receiving only one post-2015 goal, opposed to three within the eight MDGs. Less than a month later in June 2013, the Sustainable Development Solutions Network proposed its 10 SDGs [9]. Similar to the High-Level Panel, the Network proposed allocating health one umbrella goal - Goal 5 Achieve Health and Well-being at All Ages.

With apparent consensus health would receive only one post-2015 goal, from mid-2013 onward global health commentators began debating two points: what should the overarching health goal be; and, inter-related, what should comprise the content of this goal’s targets and indicators. In terms of the overarching goal, by the end of 2013 two potential contenders were apparent: UHC, particularly supported by the World Health Organization (WHO) [10]; and a ‘Healthy Life Across the Life Course’ goal (or title to similar effect), the post-2015 Global Thematic Consultation on Health’s preference [11]. The nascent debate over UHC versus a life course approach, however, highlighted a larger clash of global health and development paradigms at play.

Yet post-2015 negotiations are proving unpredictable. In April 2014, for example, WHO shifted its position by proposing a post-2015 overarching health goal ‘Ensure Healthy Lives and UHC at All Ages’ (a compromise between the so-called vertical and horizontal post-2015 factions) [12]. In July 2014, the intergovernmental Open Working Group for SDGs released its proposed list of 17 SDGs to be attained by 2030 [13]. Again, there was a standalone health goal - Goal 3 Ensure healthy lives and promote well-being for all at all ages - and the eighth of its ambitious thirteen targets called for UHC achievement. Yet Goal 3’s suggested content did not express a health and human rights framing, while the sidelining of an explicit right to health agenda was further observed in two other significant reports. First, and less surprisingly, the G77 and China’s Common Position on Means of Implementation for the SDGs of 2014 made no mention of rights [14]. Second, although the African Union’s June 2014 post-2015 report mentioned ‘rights’ nine times within that document (not completely excoriating rights like the G77 and China) [15], its Pillar 3: People-Centred Development of the Common African Position on the post-2015 development agenda (in which the health goal of ‘Universal and equitable access to quality healthcare’ is part) did not explicitly accord the right to health a central position in their suggested health goal framework.

With these three reports to hand (among others), on 10 September 2014 the UN General Assembly adopted a resolution deciding that the first of these, the Open Working Group’s outcome document, would be the main basis for integrating the SDGs into the future development agenda [16]. While the resolution stated that other inputs would also be considered during the intergovernmental negotiation process [17], it is highly likely the Open Work Group’s report with its proposed 17 SDGs (including one health goal and its litany of targets) will become the main source grounding furtive Member State mediation in 2015.

The right to health’s omission in the first half of 2014 from various intergovernmental post-MDG health and development goal proposals, and its further displacement by way of the General Assembly directive of September 2014, warrants attention as the MDG deadline nears and Member State negotiations on the post-2015 goals enters overdrive. While right to health ‘minimalists’ may argue the right to health is still in contention through the quasi-vehicle of UHC [1] [18], right to health ‘maximalists’, taking a constructivist approach, would equally contend without an explicit health and human rights narrative structuring the Open Working Group’s proposed post-2015 health and development goal, rights rhetoric will become further marginalised from ensuing Member State discussion. Maximalists would also claim that the proposal of UHC as one of 13 potential targets further eviscerates any argumentative weight and applied power the right to health may have through its UHC association.

In light of the above, there are two questions both right to health minimalists and maximalists must ask: why is the right to health not gaining effective traction in dynamic post-2015 discussion; and, what could this mean for its inherent (and ongoing) legitimacy if held separate by the Member States from the final post-MDG health goal. This article does not seek to answer the rather speculative, second question. The purpose of this analysis is to present qualitative research that addresses the positioning of the right to health in the post-2015 debate as it has temporally unfolded; research that provides crucial insight into the former question.

## Methods

**Fig.1:**
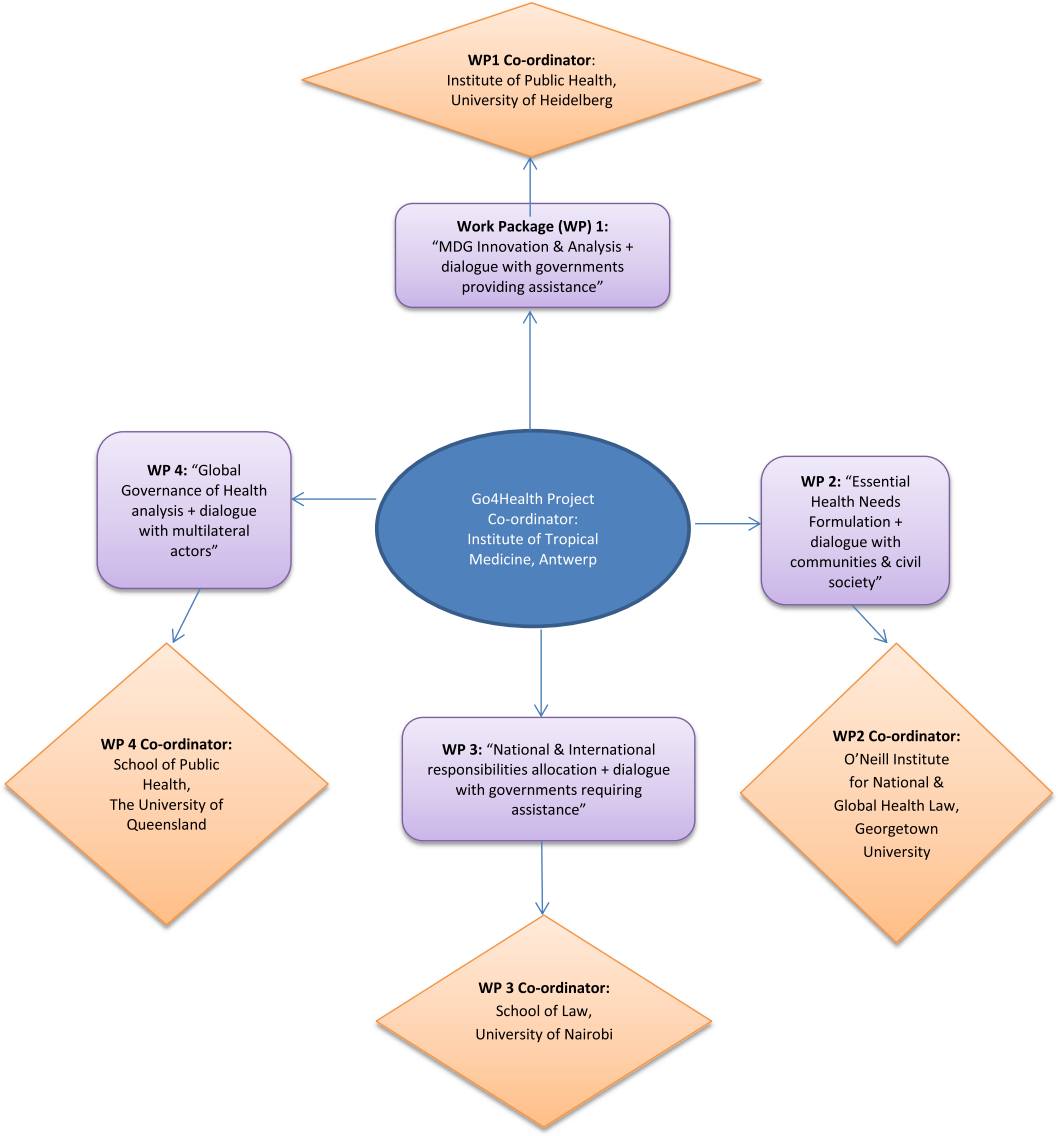
4 Go4Health’s 4 work packages and their Institutional co-ordinator

This qualitative research is part of Work Package 4 (WP4) of the Go4Health Project. Go4Health is tasked with advising the European Commission on the post-2015 global goals for health and new governance frameworks. Figure 1 identifies WP4 alongside the Go4Health Project’s three other Work Packages.

By engaging in dialogue with multilateral actors in a two-phased sequential research design, WP4 aims to both trace and investigate the emergent post-2015 Global Governance of Health landscape. In the first phase WP4 researchers (CEB and PSH) conducted semi-structured interviews with key informants recruited from multilateral and associated agencies. An inductive exploratory research design incorporating qualitative in-depth interviews was used to meet WP4’s research aims. The value of qualitative research lies in its exploratory and explanatory power, allowing researchers to gain information about an area in which little is known [19]: ideal for examining health’s location in a fluid, complex, international decision-making process.

In the first research phase, through both purposive and snowball sampling strategies, WP4 researchers selected respondents based on their identification as responsible within their organisations for health in the post-2015 development agenda (or the post-2015 development agenda more broadly). Additional informants directly linked to the health multilaterals and the post-2015 agenda from government, academia, civil society and philanthropy were also identified. Forty interviews were held in June-July 2013; 33 face-to-face interviews and seven by Skype with 57 participants, and two additional participants provided email responses. Participants were from a total of 31 agencies: 17 multilaterals, four academic institutes, three foundations, three non-government organisations (NGOs), two government agencies, and two development banks (Table 1). Broad questions were asked on the emergent post-2015 health and development goals (for a detailed outline of the pre-designed question guide, see Brolan and Hill [20]). Interviews were recorded and transcribed, with participant’s written permission (and verbal consent in some instances). Further detail on the sensitive nature of the interview process, and ensuring anonymity of the interviewees, is given in Brolan and Hill [20]. Otherwise, NVIVO 9 qualitative analysis software was used to assist coding. Preliminary analysis of the data indicated a health and human rights sub-theme among the five main themes.

**Table 1:** List of participant’s organisations, first round interviews

Agency	
World Health Organization	
Pan-American Health Organization	
UNAIDS	
The Global Fund to Fight Aids, Tuberculosis & Malaria	
GAVI Alliance	
UNICEF	
United Nations Development Programme	
United Nations Population Fund	
UN Women	
Organization for Economic Cooperation and Development’s Development Assistance Committee	
Office of the High Commissioner for Human Rights	
International Organization for Migration	
United Nations High Commissioner for Refugees	
World Trade Organization	
International Labour Office	
International Development Law Office	
Partnership for Maternal Newborn and Child Health	
UN Foundation	
Rockefeller Foundation	
Bill & Melinda Gates Foundation	
International Planned Parenthood Federation	
International Committee of the Red Cross	
Center for Global Development	
College de France	
Washington University	
The New School	
Georgetown University	
US Government	
Swedish Government	
World Bank	
Inter-American Development Bank	

Almost 12 months later in April-May 2014, WP4’s second research phase commenced with another interview round. In line with the Go4Health Project’s research agenda, these interviews focused in part on responses to Go4Health’s proposal that UHC, grounded in the right to health, be the overarching post-2015 health and development goal [1] [18]. WP4 researchers narrowed the second round interview question guide and participant sample; only interviewing participants working exclusively on the post-2015 health and development agenda in health-related multilaterals or Global Health initiatives, and specialists from NGOs and academic institutes. Second round interviews comprised 14 face-to-face semi-structured interviews with 18 participants from a total of eight agencies (five multilaterals, two academics and one foundation). Nine participants had been interviewed in the first round. The purpose of engaging this specific set of participants was to unpack the implicit tension between the Go4Health Project’s advocacy goal *vis a vis* the apparent sidelining of the right to health in post-2015 global health policy discourse, identified in Go4Health’s Work Package 3 (WP3) analysis of successive key post-2015 SDG texts from 2013 [8, 9, 11, 21].

The second set of interview data was transcribed and, similar to the data from the first round interviews, subject to thematic and discourse analysis [22–24]. Synthesis of the emerging data on a Health and Human Rights theme from both interview sets resulted in identification of six sub-themes. These six sub-themes form the findings for this paper; all contextualise why the right to health may not have gained effective traction in Member State negotiations (and evidentiary outputs) as they subsequently unfolded in 2014 after our second-round data was completed.

Ethics approval was from The University of Queensland’s School of Population Health Ethical Review Committee. The manuscript complies with BioMed Central’s research review guidelines (RATS) for reporting on qualitative studies.

## Results

This study found six reasons contextualising why the right to health may not have gained effective traction in Member State negotiations (and evidentiary outputs) as they subsequently unfolded in 2014 after our data was obtained. The first three reasons relate to broader issues surrounding human rights’ (including sexual and reproductive health and rights (SRHR)) positioning within international relations, while the second three reasons relate to the challenges of transforming the human right to health into a practically applied post-2015 health goal.

1. A key consistency: the right to health *“has been somewhat at the sides”* [Iv4178] of post-2015 health and development negotiation
  Significantly, we found just that - consensus among participants that the right to health lacks traction in evolving post-2015 health goal discussions (implicitly triangulating the content of the three later released Member State post-2015 reports and General Assembly decision of September 2014). In the second set of interviews in April-May 2014 wherein we were overt in raising the feasibility of a right to health goal, the general consensus was that the right to health *“hasn’t been picked up”***[Iv4173]** in the post-2015 health and development discourse among Member States or other key actors (such as the multilaterals). Participants were unequivocal: *“Although the right to health is mentioned, it’s not dominant in the discussion”***[Iv4170],***“I think that train has left the station around here”***[Iv4183]**, to the more direct *“the right to health is not considered as a goal”***[Iv4175]**. For a participant from one of the Global Health Initiatives, these responses were symptomatic of the marginalisation of human rights in the broader post-2015 agenda: *“the rights issue is more or less everywhere but not specifically somewhere”***[Iv4182].**
2. The right to health’s relegation is part of the broader sidelining of human rights
  Participants in both interview rounds, particularly from UN agencies, identified human rights as a potential ‘fault line’ (*“where the cracks are going to open”***[Iv1575]**) to post-2015 decision-making consensus:
  *“There are really difficult issues… child protection, rule of law, human rights… that’s a contentious issue… there’s a lot of tough discussions to be had”***[Iv1551]**
  *“Some nations … are allergic to the term ‘human rights’. They feel… it is the Western countries beating them around the head trying to impose Western cultures on them”***[Iv4172]**
  Some expressed concern that attempting to incorporate human rights language into the final post-2015 document would result in decision-making delays or *“a compromise that’s quite watered down”***[Iv1587]**. These participants anticipated the human rights agenda would be sidelined for pragmatic reasons:
  *“the [post-2015] framework [is]… going to be determined by Member States… if you start building rights language all the way through it, it’s going to take forever to get there… this is speaking on a pragmatic level… As soon as you talk about the right to that, then you’ve got all sorts of different countries arguing different things around rights as opposed to just leaving that word out. Now that doesn’t mean that it’s not the right thing to do… I just think that people take pragmatic decisions at some point about that”***[Iv4180]**
  For one participant, this pragmatism would produce highly undesirable consequences. That is, while there should be a combination of normative human rights standards within the post-2015 goal agenda, it was unlikely Member States would ultimately accede to this: *“We should be able to combine but in the end people are very literal and in countries where it’s okay to beat people, they’re just not going to sign up for that…”***[Iv1565]**. Here, “tension” existing between States’ normative responsibilities under international human rights law, and their unwillingness to translate those obligations into tangible post-2015 goals, becomes apparent. One UN participant articulated:
  *“States have committed to human rights standards and principles, but the difficulty is to now get that commitment into something like the post-2015 process, to marry the two, because they [Member States] treat it a little bit differently. Somehow there’s no synthesis between those two threads”***[Iv1574]**
  Another participant, again from the second round of interviews, considered a key barrier to marrying the *“two threads”* in the contemporary debate rested on historical factors —the formulators of the post-2015 goals’ predecessor, the MDGs, had not expressly incorporated or made adequate reference to State obligations under international human rights law (“*all the old MDGs weren’t expressed in rights’ terms”***[Iv4173]**). This had subsequently led to the emergence in the early 21^st^ century of a global development discourse that kept human rights at arm’s length. Consequently, certain actors employed this approach in the current debate, thinking “*rights are enshrined anyhow in the various constitutions, and therefore why reiterate them as goals?… They’re enshrined in the International Covenant on Economic, Social and Cultural Rights”***[Iv4173]**. This extended to the right to health:
  *“We have quite a number of international commitments to the right to health. You have the UN Human Rights Declaration, the International Covenant on Economic, Social and Cultural Rights, the WHO Constitution. How would this be different and how would you measure and how would you enforce it?”***[Iv4178]**
  Another participant expressed concern that as the United States had not ratified the Covenant on Economic, Social and Cultural Rights which contained the right to health, this in itself would *“push back”* integration of normative human rights frameworks into the post-2015 agenda, allowing many other Member States (who may have in fact ratified the Covenant) to adopt *“similar positions”***[Iv4175]**.
3. Specific anxiety around SRHR and its broader implications for negotiations
  Participants from both interview rounds foresaw Member State tension over inclusion of the phrase “sexual and reproductive health and rights” potentially frustrating incorporation of a broader human rights agenda (and thus an express right to health agenda). For a number of participants, SRHR in itself represented a ‘fault line’ to post-2015 decision-making consensus, with participants using phrases such as *“very strong antagonism”***[Iv4172]***“one of the most difficult areas for negotiation”***[Iv4170]** to describe conflicting State positions.
  However by the second set of interviews in 2014, the discourse around SRHR migrated substantially: participants were now particularly concerned SRHR were becoming increasingly connected by countries to debates around the post-2015 rights of lesbian, gay, bi-sexual and transsexual (LGBT) communities, and it was this association that was making the post-2105 SRHR negotiations *“more and more difficult”*:
  *“Rights are being made to be synonymous with gay rights of the LGBT debate, and so Member States are being much more conservative in regards to SRHR… A number of them are not okay with sexual rights”***[Iv4174]**
  *“That’s where the real sticking point will be [LGBT rights]. And if people associate a rights approach with that, then you’ve lost the Muslims and you’ve lost the African conservative countries…that’s the sort of conservative politics that you’re dealing with”***[Iv4172]**
  *“So one of the high temperature debates… is the position of the so-called LGBTs… We can’t even use that language in our discussion with Member States… it’s just too difficult to make progress on with respect to certain constituencies”***[Iv4170]**
  The association of LGBT rights with SRHR was not raised by any of the first round interviewees as a particular sticking point in post-2015 discussions: it was clear that this framing of rights was a recent, but significant emergent theme.
  While one participant mentioned how introducing a human rights agenda into the post-2015 document would be viewed by some countries as perpetuating Western imperialism, another UN participant in the second set of interviews similarly considered negotiations around the post-2015 SRHR agenda was becoming a matter of geopolitics:
  *“It’s mostly a power play between the North and South, if I can put it crudely. For instance, the Africa Group has gone very conservative and that might be the use of SRHR as a bargaining chip within the North/South debates, and the alignment of African countries with some of the BRICS priorities and so forth”***[Iv4174]**
  Respondents’ comments confirm the post-2015 debate is shaping to be a monumental time in SRHR’ evolution. On the one hand, tension exists among advocates around how to strategically preserve (and not dilute) Member State agreement on SRHR in post-2015 negotiations (and whether this involves not alluding to SRHR). While on the other hand, whether to utilise the platform of post-2015 momentum to stand up against regressive policies, emergent in conservative African States and certain power blocs.
4. An overarching post-2015 right to health goal is too big to be specifically defined
  Despite acknowledgement by some participants that the right to health was well-articulated in international law (i.e. Article 12 of the International Covenant on Economic, Social and Cultural Rights), this articulation had not translated well in (or had sufficiently grounded its presence within) the global health arena. A number of participants from the second round of interviews argued it was unclear what a right to health umbrella goal would actually mean, or how it would be defined. It lacked concreteness: *“as a goal it is really very vague actually”***[Iv4183]**; *“it’s not very explicit… [contributing to] resistance against it”***[Iv4181]**; “*quite a lot of the low-income countries find it complex language to use”***[Iv4172]**. One participant argued the right to health remains in the *“mainstream health sector and global health sector… a niche operation except perhaps the one key exception… the HIV movement”***[Iv4176]**. Another considered *“why there hasn’t been so much focus on the right to health over the last 10 to 15 years”* was part of *“a much bigger and broader discussion”*; that its marginalisation could be attributed to global health being *“more shaped and designed by economists than law or human rights”***[Iv4178]**.
5. Even if a right to health goal is defined, it is too difficult to implement
  Furthermore, several second round participants were concerned over how a right to health goal could be operationalised and practically implemented (especially with existing challenge over its definition). This query relates to participant’s parallel concern over how metrics (the measurable health targets and indicators) would or could be effectively framed by a post-2015 right to health goal. And, in most of their eyes, for better or worse, the reality was *“all the debate now is more about targets*” **[Iv4182]:**
  *“For me, it’s a very good principle, the right to health, but we need to materialise [it], really carry it out, how it spells out in different programs and different indicators. Because… it’s very hard to conceptualise what exactly we should do to get there… So people really want targets… if we don’t have a target how do we manage our program?”***[Iv4183]**
  The reticence among many of the second set of participants to endorse a right to health umbrella goal due to concern around its measurability mirrors a number of the first set of participants concerns around the challenge of integrating broader human rights into a post-2015 metrics framework:
  *“there are some things that probably can’t be measured or we’re going to have to work very creatively on some measures like governance indicators, corruption, human rights. The more softer sides of wellbeing”***[Iv1562]**
6. The right to health is already encapsulated in a post-2015 health and development goal that includes UHC, equity, or a ‘Healthy Lives Across the Life Course’ approach
  In addition to definition and implementation challenges associated with a potential post-2015 right to health umbrella goal, many participants from the second set of interviews considered the right to health on the most part was, as a principle, being conceptually or implicitly incorporated into post-2015 health and development negotiations, but not in a direct way, and prudently so. For one participant:
  *“The intellectual challenge right now is not to set a goal for the right to health…People have the right to health, full stop, the end. But the question is, how with the goals we set do we create a better assurance that people will get access to that right?… The question is, what do I need to do so that people really have the right, tangibly?… how can the whole pudding that’s going to emerge, how can that actually be more rights-based, and I think that’s what people are looking for right now.”***[Iv4173]**
  For seven participants, this tangible expression of the right to health could be found in UHC, still prominent in discussions around the post-2015 health goal, and targets and indicators:
  *“I like the conceptualisation of the right to health as the embodiment of the achieving of UHC…”***[Iv1478]**
  *“Some people are so nervous about it [the right to health] politically… I mean, it’s just like UHC”***[Iv1481]**
  Some considered a goal related to “Healthy Lives Across the Life Course” – also gaining momentum in the post-2015 health and development goal discourse - likewise elucidated the right to health (“*you could translate that into a human rights language”***[Iv4172]**). Otherwise, several considered the right to health was the same as, and/or better represented by, the use of the words ‘equity’ and ‘equality’ in post-2015 negotiations: “*when you unpack equity… you’re talking about everyone having a right… it’s often a presentation thing rather than a principle thing if you like”***[Iv4180]**; *“[the] right to health, as far as I can see – it all seems to come down to how do you address the inequalities in health in the world”***[Iv4181]**.

## Discussion and conclusions

This paper reports the views of participants from key multilaterals and related agencies on the human right to health’s location (and projected trajectory) at two temporal junctions (June-July 2013; April-May 2014) in evolving post-2015 negotiations. The interviews provide insight into high-level hesitancy that the right to health be expressly incorporated in the final post-2015 health and development goal, as well as documents participants’ doubt that human rights language will explicitly frame the broader SDG agenda. This study makes two further significant findings. First, that human rights language and normative frameworks, including the right to health, are increasingly placed at arm’s length by the health multilaterals and related agencies. Second, this is consistent with, or triangulates, emerging Member State positioning on the potential content of the post-2015 development goals (as per the content of three key intergovernmental post-2015 reports that were released in 2014 by the Open Working Group on the SDGs, the G77 plus China, and to a lesser extent the African Union).

The sidelining of human rights by Member States not only highlights the dynamism and political nature imbuing the post-2015 debate, but a shift in the locus of control in that debate from UN agencies to Member States. And, from this study’s findings, this shift also highlights how post-2015 health and development workers within the multilaterals and their allies are responding to this transition. Thus two dynamics are occurring. First, post-2015 negotiations are moving into a new locus of discourse where known country and/or regional sensitivities result in anticipatory (but selective) self-censoring of language. Second, this new pragmatics is reflected in our informant’s interactions and increasing caution around the explicit use of rights rhetoric.

Importantly, this study’s findings were later reified by events in September 2014, when the UN General Assembly endorsed the Open Working Group’s Outcome Document as the pivotal document upon which Member States were to engage in post-2015 negotiations in the year ahead. Within the suggested post-2015 health and development goal, the right to health was not mentioned, and human rights were mostly absent within the 17 SDGs and their proposed targets and indicators. Should express language around health and human rights be removed from the final post-2015 document, health rights advocates – in the spirit of social constructivism – have cause for concern. MDG experience indicates the new post-2015 health goal and targets will shape global health resources and priorities for the next fifteen years: without incorporation of express right to health language a critical communication process for domestic behavioural change in and of itself will be lost [25–27]. Moreover, there is real risk the right to health’s entrenchment in communities around the world will be stymied if not directly contained within the post-2015 health and development goal (similar to Forman’s argument in the case of the right to health and reforming trade rules on medicine [28]). Further, as right to health advocates have also contended UHC is a practical expression of this human right, its suggested positioning in the Open Working Group’s proposed health goal (the eighth of thirteen targets) evidences not only UHC’s fragility in evolving intergovernmental post-2015 discussion, but equally reinforces the right to health’s diminishing profile in the post-2015 landscape. Member State shift away from the centrality of UHC in development discussion is alarming in itself. This is given over 24 months ago (December 2012) the UN General Assembly adopted a resolution on Global Health and Foreign Policy grounded in the worldwide promotion of UHC as a decisive step in the fight against health inequality [29].

For a number of study participants, in such a complex and dynamic political environment the need for diplomatic pragmatics will likely prevail, ultimately at the expense of normative human rights. Others considered it unlikely a rights discourse could practically translate into measureable targets, or ‘value add’. For Darrow, the onus on human rights proponents to demonstrate the value-add “Rightly or wrongly… remains the dominant framing of human rights in development debates” [30]. Darrow contends a key problem concerning the “value added” challenge “stems from the comparative power of the epistemic communities within the development field, and the dominance of neo-classical economics in particular” [30]. He continues:

*“… human rights- and social rights in particular – may be disparaged as abstract or purely aspirational norms… Human rights may be categorically dismissed as inherently subjective and value-laden, compared with the putatively objective and value-neutral science of economics, and hence deserving of a particularly high standard of proof”* [30]

Darrow’s position certainly finds resonance in this study’s findings.

The right to health’s international law underpinnings act as key barriers to its express uptake in a post-2015 health goal global policy framework. Litigious potential (and ramification on States’ sovereignty and ‘bottom line’) of a right to health post-2015 goal, target or indicator creates fear that burgeoning post-2015 metrics frameworks and their ability to both contain and frame a post-2015 health goal’s borders, would be shattered. We certainly observed an anxiety among respondents to contain any migration of the post-2015 health goal framework from the quantitative towards the more challenging, politically-charged post-2015 health and development issues. Incorporation of the right to health might, for instance, allow the migration from sexual rights to LGBT rights; from the right to health to SRHR to women’s rights over their own bodies; from the right to access timely and appropriate health care (or medicines) to ambit or other claims that ‘open up the floodgates’; risking the distortion of health resource allocation and (allegedly) spelling economic disaster for country’s limited coffers. Therefore, the “pragmatic” marginalisation of human rights for many of our respondents was most apt as a means to thwart such future apocalyptic imaginings, and certainly most economically prudent.

Conversely, what right to health doubters fail to comprehend is while normative international human rights laws (as well as the justiciable potential they present) and metrics frameworks may appear to operate distinctly and separately when it comes to responding to health and inter-related post-2015 inequities, neither approach alone is sufficient to achieve the post-2015 goals and the significantly broadened environmental and human development agendas they will likely contain [31, 32]. Thus, rather than creating a transformative post-2015 agenda, by continuing the MDG route of divorcing rights from development goals there is real risk the post-MDG agenda will be anything but transformative – but regressively reduced to meeting basic needs, to meeting (even more) targets and sub-targets, as opposed to overcoming in-country development inequities.

### Study Limitation and Strength

This study’s central limitation is the question guide for the first interview round did not pose specific questions on the right to health’s intersection in the post-2015 agenda (or human rights more broadly). Thus this study could only rely on the responses of 17 (or 33 %) of participants from 14 (or 35 %) of the 40 first round interviews held. Possibly our results may have differed, and certainly been enriched, had all first round participants been systematically questioned about a post-2015 human rights intersection. It follows that the six reasons participants gave why rights are missing from SDG debate cannot exhaust the total number of possibilities, but certainly reflect some major reasons requiring further examination.

That said, this study’s central limitation could be interpreted as a strength: The lack of canvassing on the right to health in the first data collection round is in-keeping with WP4’s exploratory, inductive research design. Certainly, in the second research phase, it was apposite WP4’s participant sample and interview guide altered: the sequential in-depth interviews were not two simultaneous exercises, but social research designed to follow the “temporal rhythm” [33] of an evolving, live decision-making phenomenon; a macro-level policy discourse on health and the post-2015 development goals.

Despite risk of being accused of academic presumptuousness, we submit this study’s historical significance should not be downplayed. When in 2009 David Hulme investigated the “processes that led to the specification and agreement on the MDGs” he highlighted:

“[e]xamining these processes is fundamental to understanding why the MDGs have their present content and structure, and may offer practical insights to those involved in MDG implementation seeking to influence future global mega-promises (and there will be more of them)” [34].

The time for another global mega-promise, as Hulme foresaw, has arrived. Unlike the vacuum of contemporaneous analysis as to ‘why’ the health MDGs (MDGs 4–6) were incorporated into the MDG ‘list’ of 2000 (indeed, Hulme’s analysis is a reflexive study) - or, more exactly, ‘what happened’ to health and human rights (and SRHR’s) marginalisation from the original MDG ‘list’ in 2000 - this research provides contemporaneous evidential fodder for future rights’ activists and academics tracing the temporal journey of not only the right to health, but broader questions related to the very fluid global valuing of economic, social and cultural rights.

## Abbreviations

High Level Panel: High-Level Panel of Eminent Persons on the Post-2015 Agenda
MDG: Millennium Development Goals
NGO: Non-government organisations
SDG: Sustainable Development Goals
SRHR: Sexual and reproductive health and rights
UHC: Universal health coverage
WHO: World Health Organization
